# Danger Signals in Nitrous Oxide Oxygen Anaesthesia1Read before the Winnipeg Dental Society, March, 1921.

**Published:** 1921-11

**Authors:** E. Roy Bier

**Affiliations:** Winnipeg


### DANGER SIGNALS IN NITROUS OXIDE OXYGEN ANAESTHESIA [1]

DR. E. ROY BIER, WINNIPEG

All attempts to cover the Nitrous Oxide and Oxygen administration in so short a time would be time wasted, and so I will not detain you, gentlemen, very long, but will endeavor to point out the danger signals, and how to overcome them, as well as to throw some light on a few relative points.

The father of skill is industry, and anyone desiring to become an Anaesthetist may do so, provided he keeps at it, working industriously to understand the normal and abnormal symptoms and learning to differentiate between them.

Nitrous Oxide and Oxygen Anaesthesia is so rapidly induced that, unless you can positively understand when overdosage for that particular patient has started, death may result much more rapidly than by using any other anaesthetic.

The Committee on Research of Anaesthetics in the U. S. A. brought in this report: that Nitrous Oxide and Oxygen was the safest anaesthetic agent in the world at the present time, in competent hands, and the most dangerous agent in the hands of the incompetent. Today in Winnipeg, not half a dozen medical men in the city are allowed by the hospital boards to administer it for major operations. Just recently, when I had my appendix removed, I took Nitrous Oxide and Oxygen. I told the superintendent what anaesthetic I preferred, and was told that only one man in that hospital was allowed to administer it. I then made enquiry why so much caution in the use of Nitrous Oxide and Oxygen, and they said it was due to past accidents with Nitrous Oxide and Oxygen, and its

[1] Read before the Winnipeg Dental Society, March, 1921.

difficulty in administration. Surely, then, the danger signals are far the most important I could speak of.

First of all, use a pure gas—tested by a reputable chemist. Then before using each new cylinder, smell the gas and see whether it has the sweet, pleasing smell of Nitrous Oxide or a sharp pungent smell of impurities which may be due to an unwashed cylinder or a trace of chlorine or nitric oxide.

Use a machine easy of manipulation and one where percentages are controllable by a definite knowledge.

Use Oxygen from the start of the anaesthetic with all patients, except plethorics, varying the percentage according to the needs of the patient. This will oxygenate the circulation and the tissues, as well as the respiratory organs, and death from asphyxiation is not apt to happen nearly as rapidly. One should be able to recognize:

1. Heart lesions and circulatory disturbances, also take blood pressures.
2. Respiratory disturbances. Look for:
    (a) Enlarged tonsils.
    (b) Hypertrophied polypii.
    (c) Swollen mucous membranes.
    (d) Tuberculous condition.

The sound of the breathing should be so familiar to the Anaesthetist that he would almost be able to look out of the window and tell what stage of anaesthesia his patient is in from the sound of the breathing. The more nearly it resembles a natural sleep with a faint snoring sound, which is due to the vibration of the soft palate, the nearer perfect is your surgical stage of anaesthesia, and any deviation from that means a deviation from the normal or perfect anaesthesia. Very shallow, light breathing, hardly noticeable, means one of two things—over-narcosis, approaching very dangerous stage, or under-narcosis, coming out of the anaesthetic. If your patient is overdosed, no doubt, cyanosis will be present. Remove the inhaler immediately shut off all Nitrous Oxide, and administer Oxygen, if necessary. If the color is very pink, in all probability the patient is coming out of the anaesthetic, and you must shut off the Oxygen and more deeply anaesthetize the patient.

If pale and sweating, remove inhaler and give Oxygen All questions of focal infections today are just a question of dealing with the longevity of our patients (their health and comfort, of course, contribute to their longevity), and every time one gives a general anaesthetic we are dealing with the life of that patient. No matter what contribution our skill and work in whatever branch of dentistry we are engaged in, we are dealing with health and comfort of the patient, and for these gifts of skill we should in return command the respect and remuneration of our patients according to services rendered.

Over-narcosis means destruction of haemoglobin and the erythrocytes. Casto, of Philadelphia, has shown the destruction of red blood cells to be as much as 16 per cent. from overdoses in major operations. His experiments were done on animals, as well as man.

Destruction of red blood cells means the system of the patient is so much weaker. These red blood cells have to be replaced and again manufactured and placed in the blood streams. That patient has not as high a resistance to fight against disease, and post-operative recovery is lowered. However, that destruction of red blood cells can be largely overcome by using Oxygen from the start of the administration and keeping the patient in normal anaesthesia. Warming the anaesthetic agent is also a safeguard toward danger of producing undue excitement which is at times caused by a cold agent, which irritates respiratory passages and reflexly irritates the brain, causing excitement.

In experiments on animals with cold anaesthetics, death was accompanied by convulsions. Similar experiments with warm anaesthetics showed death was more tranquil.

Lung hemorrhages in tubercular patients may be avoided by using a large percentage of Oxygen and warming the Nitrous Oxide during administration. It has been proven that warm Nitrous Oxide is less irritating to mucous membranes and causes less swelling, less stimulation to salivary glands, less mucous, hence less nausea.

Warming the agent. Warm gases at body temperature are more normal, and pre-existing gastro-intestinal disturbances are often prevented, because acidosis and cellular changes are entirely eliminated. This, therefore, lessens the danger of after effects and removes the danger of anaesthetic shock, and is considered a safeguard of life generally.

Recovery from a warm vapour is much quicker during prolonged anaesthesia than from a cold vapour.

Gwathmey says experiments on humans result in rapid recovery by passing warmed air into the lungs after ether anaesthetic or any anaesthetic, and putting hot towels about the patient's face, and delayed after effects are reduced to a minimum.

*Rebreathing* is a very important factor. 1. Acapnia[2] results from the lack of rebreathing, which means a loss of Carbon Dioxide in the blood. Carbon Dioxide is the normal respiratory stimulant, and when an open method is employed the Carbon Dioxide is lost.

Henderson has been able to reduce animals to a state of shock by over-ventilation and getting rid of Carbon Dioxide in the body.

*Rebreathing*—2. Lessens post-anaesthetic vomiting. 3. Decreases the number of cases of abdominal distention. 4. Reduces post-anaesthetic lung complications. I have anaesthetized 2,000 cases of lung complications, bronchitis, pulmonary T.B., empyema of the lungs, without hesitation,

---

[2] Acapnia only develops in long anaesthesias.

and only two cases had any reactions and no deaths occurred.

Deaths under anaesthesia from Alcoholics precipitate in the following symptoms:

1. Ascending degree of cyanosis.
2. Increased excitation.
3. Tonic or chloric spasms of the musculature, with embarrassment of respiration, asphyxia and abrupt cardiac arrest.
4. Over-ventilation, acapnia, pallor, apnea, gradual cardiac exhaustion.

Alcoholics and dope fiends are bad risks, as chronic alcoholism, according to Dr. Mechan, causes the following pathology:

1. Arteriosclerosis and Cardiac Hypertrophy.
2. Increased inter-cranial pressure and edema of the brain.
3. Cirrhosis and fatty degeneration of the liver.
4. Increases susceptibility to post-operative infection.

The men who achieve success are those who have worked, thought and read more than is necessary, who have stored knowledge for an emergency reserve. It is superfluous work that equips a man for everything that counts most in life. The art of anaesthesia is not contained in any one man's teaching of a certain method of administration; it is the knowledge gathered with the skillful observation of human reaction to different anaesthetic agents, and a broader sense of mental and physical equilibrium of the patient. The art of anaesthesia is an intuition developed by experience.

Much of the psychic fear of a patient depends upon the confidence or lack of confidence a patient places in the anaesthetist, and when a patient so lacks confidence, that is the first step towards the production of shock.

Blood pressure is important because it gives early warning of the presence of shock. It may uncover: Arteriosclerosis, Nephritis, Myocarditis, Aortic insufficiency, Mitral Stenosis or anaesthetic depression.

Premedication absolutely advisable for above.

SHOCK

*Surgical*—Manipulation by surgeon, loss of blood.

*Anaesthetic*—Overdosage or too light anaesthetic, or obstructed airway.

*Psychic*—Due to the powerful impulses from highly specialized centres of the cerebrum acting upon the vital centres of the medulla.

*Dietetic*—Due to preliminary fasting before and after operation, abdominal cases.

*Toxic*—Abscesses with pus running down throat, absorption of chemical irritations reflexly. The patient requires larger doses of Oxygen or withdrawal of anaesthetic and cessation of operation. Fall in blood pressure always precedes shock by several minutes, sometimes as long as half an hour in ether narcosis.

*Anoci-association*—(Use of general and local anaesthetics—to obtund sensations from reaching the brain)—Preliminary medication of narcotics, morphia or chloretone to keep patient from any emotional excitement. Not to allow patient to worry about the date or time of operation, have patient put into hospital, give Nitrous Oxide to them while they are asleep, then use local anaesthetic and block the field so completely that no traumatic (cutting, stretching or pulling) impulses reach the brain. After the wound is closed again block with a local anaesthetic of Quininurea, preventing after pains for twenty-four hours. Thus the motor mechanism has received no adequate stimulus, and there is no surgical shock, no interference with digestion, no nervous impairment afterwards, no change in the circulation, respiration, digestion or the mentality of the patient.

Respiratory paralysis is caused by:

(a) Blood clot in throat.
(b) Swallowing tongue.
(c) Pieces of tooth.
(d) Throat packs.

Precautions:

1. Keep open airway.
2. Mechanical forcing of oxygen into lungs.
3. Throwing forward of mandible keeps open airway and reflexly stimulates the nervous system, maintaining anaesthesia.
4. Massage abdomen over viscera from pelvic region upwards. Internal abdominal pressures, enemas, opening of sphincter muscles.
5. Artificial respiration.
6. Inversion:
    (a) Lowering head.
    (b) Complete inversion.

Keep blood in brain, so as not to produce an anaemic condition of respiratory centre, thereby prolonging the action of respiration. Swinging should be done vigorously.

*Inversion*—Grasp by the knees and swing to and fro. This distends the heart and intercranial vessels. Also, the diaphragm presses down, producing a larger air space, causing a vacuum and the A.T. pressure of 14½ pounds per square inch. The outside air rushes into patient's lungs. Swinging may force it out, then we have a mechanical bellows which will keep up respiration if our airway is clear to allow the passage of air into the lungs. If airway is blocked, have the assistant pound the patient on the back while you are swinging the patient. If you can not obtain open airway, stretch sphincter muscles of ani, and swing in inverted position. If this does not produce results, do a Trachaetomy.

7. *Drugs:*

(a) Camphor and oil.
(b) Whiskey.
(c) Aromatic Spirits of Ammonia.
(d) Amyl Nitrite.
(e) Units of Strychnine, etc. —*Oral Health.*

